# Antigen dose and humoral immune response correspond with protection for inactivated infectious pancreatic necrosis virus vaccines in Atlantic salmon (*Salmo salar* L)

**DOI:** 10.1186/1297-9716-44-7

**Published:** 2013-02-11

**Authors:** Hetron Mweemba Munang’andu, Børge Nilsen Fredriksen, Stephen Mutoloki, Roy Ambli Dalmo, Øystein Evensen

**Affiliations:** 1 Department of Basic Sciences and Aquatic Medicine, Norwegian School of Veterinary Science, PO Box 8146 Dep.,N-0033 Oslo, Norway; 2 Faculty of Biosciences, Fisheries & Economics, University of Tromsø, 9037 Tromsø, Norway

## Abstract

An enduring challenge in the vaccinology of infectious pancreatic necrosis virus (IPNV) is the lack of correlation between neutralizing antibodies and protection against mortality. To better understand the immunological basis of vaccine protection, an efficacy trial including Atlantic salmon (*Salmo salar* L.) vaccinated with a high antigen (HiAg) or low antigen (LoAg) dose vaccine was carried out in a cohabitation challenge model using the highly virulent Norwegian Sp strain NVI015. To pinpoint the immunological basis of vaccine protection, pathogenic mechanisms of IPNV were unraveled in control fish while obtaining feedback on mechanisms of protection in the vaccinated fish. During the incubation period, infection rates were highest in control fish, followed by the LoAg group with the lowest infections being in the HiAg group. Although both the liver and pancreas are target organs prone to tissue damage, infection in the liver was delayed until acute infection in most fish. A correlate of pathology determined as the cutoff threshold of viral copy numbers linked to tissue damage in target organs was estimated at ≥ 10^7.0^, which corresponded with an increase in mortality. The kinetics of IFNα and Mx expression suggests that these genes can be used as biomarkers of IPNV infection progression. Mechanisms of vaccine protection involved reducing infection rates, preventing infection of the liver and reducing virus replication in target organs to levels below the correlate of pathology. Overall, the study shows that antigen dose corresponds with vaccine efficacy and that antibody levels can be used as a signature of protective immunity against pathological disease and mortality.

## Introduction

Infectious pancreatic necrosis virus (IPNV) is a highly contagious disease causing high mortality in juvenile salmonids. The disease was first reported as catarrhal enteritis mainly affecting fingerlings in North America in the 1940s [1] although the virus was first isolated and characterized in 1960 [2]. It is a double stranded RNA virus and a prototype member of the *Aquabirnavirus* in the *Birnaviridae* family [3]. The expansion of aquaculture which has led to intensive fish farming has resulted in an increase in outbreaks not only in fingerlings but also in postmolts [4]. To reduce the occurrence of outbreaks, research in the last two decades has focused on finding the most protective, ecosafe and cost effective vaccines able to reduce mortality and post challenge persistent infections [5-8]. Although several strategies have been explored to develop efficacious vaccines, success has been limited by the general failure of most vaccines to produce long lasting immunity that reduces mortality and eliminates post challenge persistent infections.

Factors leading to vaccine failure have been reviewed by different scientists. In 1986, Olesen and Jogensen [9] reported that antibody responses in salmonids were inherently variable and that they could not be correlated with protection. A subsequent study carried out by Bootland et al. [10] in 1990 showed that inactivated vaccines were able to reduce mortality but did not prevent infection. This is in line with several other studies that reported of the coexistence of infecting virus and circulating antibodies. In a more recent study, we delivered IPNV VP2 as a fusion protein in *Escherichia coli-*based subunit vaccines, plasmid DNA encoding segment A, PLGA nanoparticles and inactivated whole viral vaccines (IWV) in water-in-oil formulation in Atlantic salmon [11]. Overall, IWV were superior but a protective proportion of 56% for the most protective vaccine was considered low and this was coupled with the general failure to eliminate post challenge persistent infections. These findings still confirm observations from previous reports showing that vaccination can reduce mortality but fail to eliminate infection in survivors. Although different scientists [12-20] have reviewed the efficacy of different IPNV vaccines, less attention has been directed at elucidating the functional mechanisms of vaccine protection in fish. What is eminent from these studies is that they lack detail on mechanisms of vaccine protection because they do not pinpoint the exact mode of action by which IPNV vaccines protect fish from dying. In the absence of such detail it is difficult to fully account for factors that lead to vaccine failure.

Unlike in IPNV vaccinology, vaccine development in higher vertebrates has taken a more functional approach. In mammalia, vaccines are licensed based on a good understanding of mechanisms of protection and defined correlates of protection [21]. Although several vaccines have been licensed, there is no established correlate of protection for IPNV vaccines. Apart from determining the relative percent survival (RPS) as a measure of efficacy, the exact mode of protection induced by these vaccines has not been determined. It is not clear whether vaccination prevents virus penetration at portals of entry, dissemination, seeding in target organs or tissue damage in target organs. Besides, there is no established standard antibody titer that corresponds with protection. In view of these gaps, a vaccine efficacy trial was designed using fish vaccinated with a high antigen (HiAg) and a low antigen (LoAg) dose vaccine, put to cohabit with unvaccinated control fish. The study sought to unravel the pathogenic mechanisms of IPNV in control fish while getting feedback on mechanisms of protection in vaccinated fish. Hence, the study was aimed at (i) documenting whether antibody responses at the time of challenge correspond with protection against mortality, (ii) determining tissue tropism, the pathogenic sequence of infection and to what extent vaccines interfere with pathogenesis, and (iii) identifying potential biomarkers that correlate with progression of infection.

## Materials and methods

### Viruses and cells

A highly virulent recombinant IPNV strain produced by reverse genetics using cDNA from the Norwegian Sp strain rNVI015 (Genbank: AY379740) [22] was used as antigen for the production of the vaccine and in all immunoassays carried out during the study. The virus was also used to challenge the fish. Propagation of the virus was done in Rainbow trout gonad (RTG-2) cells maintained in Leibowitz L-15 media (Gibco® Life Technologies, Palsey, UK) supplemented with 10% fetal bovine serum (FBS), 10% L-glutamine and gentamycin 50 μg mL^-1^.

### Vaccine formulations

Vaccine antigens were made by growing the highly virulent Norwegian strain rNVI015TA once in RTG-2 cells followed by another passage in Asian Group-strain K cells (AGK) [23]. Viruses were harvested when a total cytopathic effect (CPE) was observed from both cell lines. The AGK cell line gave yields of approximately 2 × 10^10^ TCID_50_/mL. Thereafter, two vaccines were constituted as a high antigen dose (HiAg) with 2 × 10^10^ TCID_50_/mL and a low antigen dose (LoAg) with 2 × 10^9^ TCID_50_/mL and were administered at 0.1 mL/fish. The vaccines were prepared as oil-based inactivated (water-in-oil) formulations. Emulsification of the viral antigen with adjuvant was done using a homogenizer with a standard emulsification stator/rotor connected to an emulsion screen. The oil-based antigen preparation was formulated as water-in-oil (w/o), where the water phase (containing viral antigens) was dispersed into an oil phase (continuous phase containing emulsifiers and stabilizers). Vaccine preparation and quality assurance were all carried out by PHARMAQ AS, Oslo.

### Experimental fish and study design

**Figure 1:**
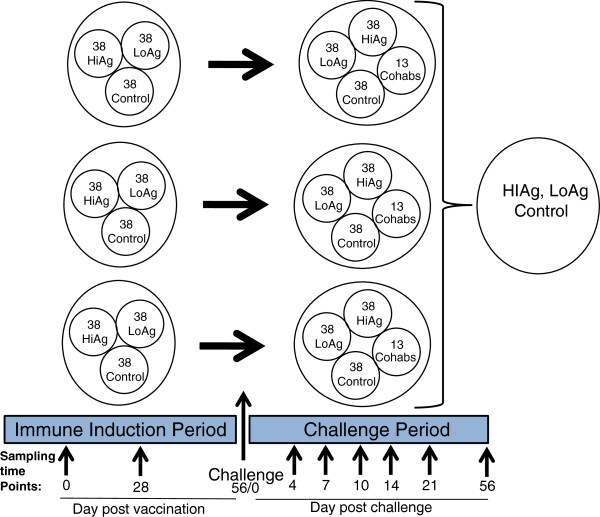
**Study design showing a challenge model based on a three parallel tank system.** Each of the vaccine groups was allocated 38 fish while the unvaccinated control group had 30 fish in each tank. At day 56 (8 weeks) post vaccination, fish were challenged with a highly virulent Norwegian IPNV strain NVI-015TA (AY379740) using a cohabitation model. In each tank, 13 fish injected with 1 × 10^7^ TCID_50_/mL of the challenge virus were added to serve as virus shedders.

Fish experiments were carried out at the Aquaculture Research Station at the University of Tromsø, Norway, using susceptible Atlantic salmon (*Salmo salar* L). Atlantic salmon parr with an average weight of 30 grams were used in the study. Fish were assigned into three parallel tanks and were fed commercial dry pellets (Skretting AS, Stavanger, Norway) *ad libitum* using automated equipment while water in the tanks was pre-treated with UV-light. Each vaccine group was allocated a total of 114 fish that were split into 38 fish per tank (Figure 1). For identification, fish were intraperitoneally implanted with pit-tag-numbers using a tag applicator. Tag numbers were entered in an excel sheet (Microsoft Excel™) using an automated electronic reader (ARE-H5-150, Trovan Ltd, Koln, Germany) remotely connected to a computer. Vaccination was carried out by intraperitoneally injecting each fish with 0.1 mL of the vaccine. The control group was allocated a total of 90 fish of which 30 were put in each of the three parallel tanks (Figure 1). Control fish were injected with 0.1 mL/fish of phosphate buffered saline (PBS). During tagging, vaccination and sampling, fish were anaesthetized using 40 mg benzocaine per liter of water (Benzoak Vet., ACD Pharmaceuticals, Alesund, Norway). After vaccination, fish were subjected to a light regime to induce smoltification during the immune induction period. After eight weeks post-vaccination, fish were challenged in a cohabitation model by adding 13 virus shedders in each tank (Figure 1). The virus shedders were intraperitoneally injected with 0.1 mL of the homologous virus (rNVI015TA) to the one used in vaccine preparation at a concentration of 1 × 10^7^ TCID_50_/mL.

### Sampling

Pre-challenge sampling was carried out on days 28 and 56 post vaccination. At 4, 7, 10, 14, 21 and 56 days post challenge (dpc) more samples were collected as shown in the study design in Figure 1. The samples collected included head-kidney, spleen, gills, heart, muscle, liver and pancreas in RNA-later and 10% formalin. Head kidney samples were also collected in transport medium consisting of L-15 maintenance media (section “Viruses and cells”) without FBS but with 30% glycerol while blood samples were collected in EDTA tubes and stored at −20°C.

### Post challenge virus re-isolation

Head-kidney samples collected in transport media were homogenized and 100 μL was inoculated onto confluent RTG-2 cells at a dilution of 1:10 and 1:100. The inoculated cells were incubated at 15°C for seven days. Thereafter, the supernatant was passaged onto new confluent RTG-2 cells and observed for another seven days. The presence or absence of virus was scored on the basis of cytopathic effects (CPE). Similarly, 100 μL of whole blood stored in EDTA vacutainer tubes was inoculated on confluent RTG-2 cells. The same procedure of screening as described above was followed.

### ELISA

Briefly, 100 μL per well of polyclonal rabbit anti-IPNV (K95) [24] was coated onto 96 well micro-titer plates (Nunc Maxisorb, Roskilde, Denmark). Thereafter, the plates were placed at 4°C overnight. After washing, 250 μL of 5% dry milk in PBS/T was added to each well followed by incubation at room temperature for 2 h. After washing, 100 μL IPNV supernatant was added to each well and the plates were incubated for 2 h. Test sera diluted in a twofold dilution starting at 1:50 together with plasma controls, blank (1% dry milk PBS/T) and IPNV positive control sera were added to the wells on the plate after washing. Thereafter, plates were incubated at 4°C overnight. After washing, an anti-salmon mouse monoclonal antibody (4C10) [25] was added to each well followed by incubation for 1 h. A goat-anti-mouse antibody conjugated to a horseradish peroxidase (DAKO; Glostrup, Denmark) was added to each well after washing. This was followed by adding 0.1 mL OPD substrate (O-Phenylenediamine dihydrochloride, DAKO; Glostrup, Denmark) containing 30% H_2_O_2_ to each well. Color development was stopped by adding 0.05 mL H_2_SO_4_ per well. The results were read using an ELISA reader spectrophotometer (TECAN, Genios, Boston, USA) at a wavelength of 492 nm.

### Histology and immunohistochemistry (IHC) staining

The technique described by Evensen and Lorenzen [26] was used for immunohistochemistry (IHC) staining. Briefly, after deparaffinization and hydration, tissue sections were blocked with 5% bovine serum albumin (BSA) in 1M Tris-buffer pH 7.6 for 20 min. After washing, anti-IPNV polyclonal rabbit antisera (K95) diluted in 2.5% BSA was added to each slide. After incubation for 30 min a secondary biotinylated anti-rabbit antibody (Dako, Glostrup, Denmark) was added after washing. This was followed by adding streptavidin alkaline phosphatase (Sigma, Aldrich, St Louis, USA) diluted in 2.5% BSA in tris-buffer after incubation for 30 min. After washing, fast red substrate (Sigma, Aldrich) was added to each slide and observed for 15 min. The reaction was stopped by washing the slides in tap water followed by counterstaining using hematoxylin dye for 2 min. Thereafter, slides were observed under a light microscope. The histology was carried out as described elsewhere [27].

### RNA extraction and cDNA synthesis

Total RNA from head-kidney, pancreas, liver and spleen organs was extracted using a combination of the Trizol® (Gibco® Life Technologies, Palsey, UK) and RNAeasy Mini kit (Qiagen, Hilden, Germany) methods. Briefly, 30 mg of tissue was homogenized in trizol and spun at 12 000 × *g* for 10 min at 4°C. Thereafter, 0.2 mL of chloroform was added to each vial followed by vortexing for 15 s. The samples were incubated at room temperature for 5 min followed by spinning at 12 000 × *g* for 15 min. The upper aqueous phase was transferred to new vials and mixed with 0.6 mL 70% ethanol by vortexing. Sample solutions were transferred to RNeasy spin columns placed in 2 mL collection tubes. Thereafter, the Qiagen protocol was used according to the manufacturer’s recommendations. The quality of purified RNA was assessed by running the samples on 1% agarose gel and the samples were quantified using a spectrophotometer (NanoDrop® ND-1000, Thermo Scientific Inc, Wilmington, USA).

The synthesis of cDNA was carried out in 20 μL reaction volumes using the quantiTect® reverse transcription method (QuantiTect®, Qiagen, Hilden, Germany). Briefly, 2 μg gDNA wipeout buffer (7×) and 1 μL of RNAse-free water were added to 1 μL of template RNA. After incubation at 42°C for 2 min, 1 μL of quantiscript reverse transcriptase, 4 μL quantiscript RT buffer (5×) and 1 μL RT primer mix were added to the template solution on ice. The incubation steps were carried out in a thermocycler (Bio-RAD, DNA Engine, Peltier Thermal Cycler, Hercules, California, USA) at 42°C for 15 min followed by a second step at 95°C for 3 min. The cDNA was diluted 1:5 using RNAse free water and was stored at −80°C until use. Two microliters were used for each reaction in the Syber green qPCR assay (Roche, Mannheim, Germany).

### Construction of the standard curve

A plasmid (pUC19) construct containing segment B of the SP strain NVI015 [28] was used as template for generating the standard curve used for quantifying virus in infected tissues. Transformation of plasmid DNA was carried out in *Escherichia coli* competent cells (Qiagen Hilden, Germany). Thereafter, linearized plasmid DNA was quantified using a spectrophotometer (NanoDrop® ND-1000, Thermo Scientific Inc.). Plasmid DNA was serially diluted from 10^9^ to 10^1^ and diluents were used to estimate the number of viral copies using the master hydrolysis probe method (Applied Biosystem, Warrington, UK). All reactions for quantitative PCR (qPCR) were constituted as 20 μL volumes consisting of 2.0 μL diluted plasmid DNA template, 2.0 μL primers, 1.3 μL Activator (50 mM), 7.4 μL Lightcycler® 480 RNA master hydrolysis probe (2.7×), 1.0 μL enhancer (20x) and 6.3 μL dH_2_O (PCR-grade). qPCR was done using the LightCycler® 480 thermocycler (Roche Diagnostics, Mannheim, Germany) in 96 multiwell plates (Roche Diagnostics). Copy numbers were calculated as described by Godornes et al. [29] while the standard curve was generated by plotting the C_t_ values on the Y axis and the log of copy numbers on the X-axis.

### Quantification of virus

RNA from headkidneys, spleen, liver and pancreas were used as template for the quantification of virus. The same primers and probe used for the standard curve were also utilized to quantify virus in these organs. A one-step kit (Roche Diagnostics) was used for this purpose in the LightCycler® 480 thermocycler with the following conditions: 63°C for reverse transcription, 95°C for 30 s to stop the RT reaction and initial denaturation; 95°C for 10 s, 60°C for 30 s and 72°C for 1 s for qRT- PCR. Quantification of viral copy numbers in test samples was estimated by relating the C_t_ values obtained in the samples to the standard curve generated using plasmid DNA according to the method described by Yu et al. [30].

### Real time PCR for innate genes

Real time PCR using the SYBR® green (Roche) detection method was used to determine the expression of IFNα and Mx genes in different organs. Primers for these genes as well as the house-keeping genes β-actin and ELF-α are presented in Table 1. RT-PCR amplification cycles were carried out in a Light-Cycler® 480 machine (Roche Applied Science). qPCR reactions were constituted and performed as described above with the following cycling conditions: 95°C for 10 min initial denaturation; 95°C for 3 s, annealing temperatures for 10 s, 72°C for 30 s (40 cycles). The melting curve analysis was done at 95°C for 5 s and 65°C for 1 min. Transcription levels for the target genes were quantified relative to internal control genes using the delta-delta method [31,32].

**Table 1:** Primers used for quantitative PCR

**Gene**	**GenBank**		**Sequence 5´-3´**	**Tm (°C)**	**Size (bp)**
β-actin	BT047241.2	F	CCAGTCCTGCTCACTGAGGC	63.5	75bp
		R	GGTCTCAAACATGATCTGGGTCA	63.5	
ELFα	AF321836	F	GCTGTGCGTGACATGAGG	58.2	88bp
		R	ACTTTGTGACCTTGCCGC	58.2	
Mx (1)	SSU66475	F	TGCAACCACAGAGGCTTTGAA	57.9	78bp
		R	GGCTTGGTCAGGATGCCTAAT	57.9	
IFNα (2)	NM_001123570	F	TGGGAGGAGATATCACAAAGC	57.9	163bp
		R	TCCCAGGTGACAGATTTCAT	57.9	
ProbeIPNVsegB	AY379740		6FAM-CCGGATTCCTAGACGAC		
		F	GACTGGAGGTAAAAGGCATCGA	60.3	68bp
		R	CCGAACTCCGACATGGTGTT	59.4	

### Statistical analysis

Survival analysis was used to generate survival plots and to determine the proportion hazard risk ratios for the different vaccinated groups using p/c mortality data. Analysis of Variance (ANOVA) was used to determine the difference in antibody response between the different vaccine groups when compared to the unvaccinated control. The Pearson Chi-square test was used to determine the correlation between mortality and the presence of disease.

## Results

### Post challenge survival proportions (PCSP)

**Figure 2:**
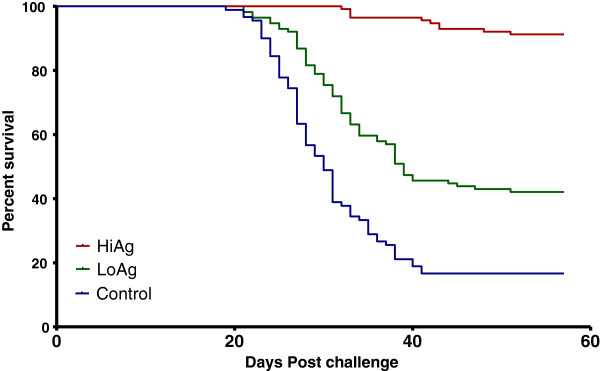
**Kaplan Meyer’s post challenge survival proportion (PCSP) plots for the vaccinated and unvaccinated-control groups.** The highest PCSP was in the HiAg group followed by the LoAg group and was the lowest in the unvaccinated control group.

Clinical signs of irregular swimming and distention of the abdomen were observed in affected fish. Daily mortality data of fish collected from each tank was used to generate the PCSP and the Kaplan Meyer’s survival plots. Among the virus shedders, mortality started on day six p/c. The cohabiting fish started dying 19 dpc in the control groups while in the LoAg group they started dying 21 dpc and in the HiAg group mortality was delayed until 32 dpc (Figure 2). There was a significant difference in mortalities between vaccinated fish and controls (*p* < 0.0001 for both HiAg and LoAg). The risk of fish dying in the LoAg group relative to the most protected group (HiAg) was 9.38 while the risk of unvaccinated fish in the control group relative to the HiAg group was 21.05 (Table 2). Onset of mortality was dependent on antigen dose; mortality in the HiAg started 11 days after the LoAg group while fish in the control group started dying two days earlier than in the LoAg group. Overall, vaccine efficacy corresponded to antigen dose, as shown by the high dose of 2 × 10^10^ TCID_50_/mL used in the HiAg, which was protected by PCSP > 90% compared to the low dose of 2 × 10^9^ TCID_50_/mL used in LoAg that had low protection (42.1%). Amongst the controls, 15.4% of the fish survived.

**Table 2:** Proportion of hazard risk ratios for fish vaccinated with the LoAg vaccine and unvaccinated control fish challenged with Sp strain NV015

**Vaccine group**	**Hazard risk**	**Std err**	**P > |z|**	**95% Conf. Interval**
LoAg	9.384	3.190	0.000	4.820 – 18.271
Cntrl	21.056	7.147	0.000	10.826 -40.952

Hazard risk ratios are expressed relative.

### Antibody responses

**Figure 3:**
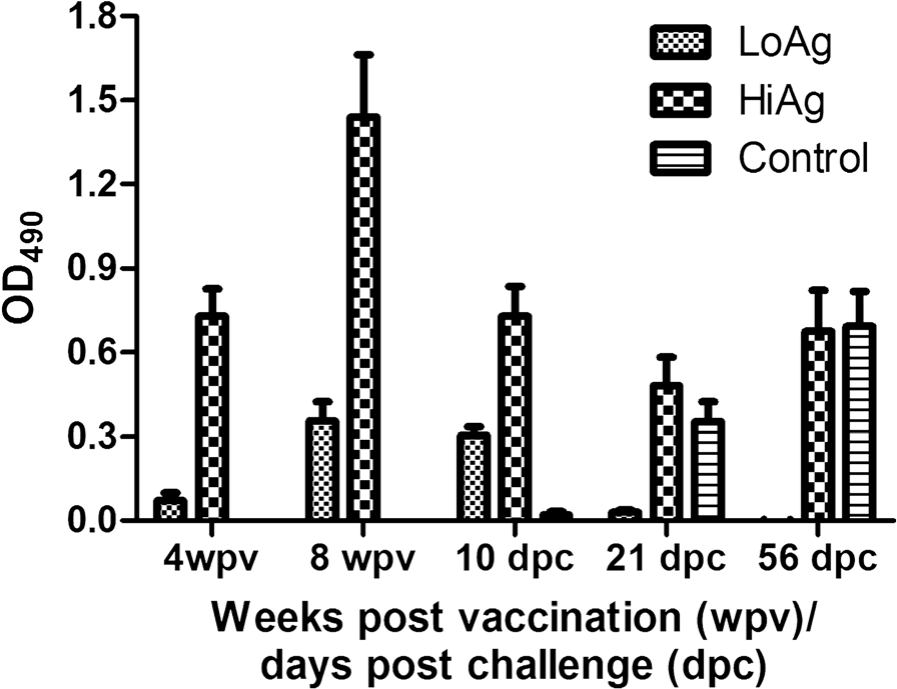
**Antibody levels detected by ELISA.** Four weeks post vaccination (wpv) up to 8 wpc, which is equivalent to day of challenge, shows antibody responses generated during the immune induction period. Days after 8 wpc show antibody levels after challenge. There is a marked decline in circulating antibody levels post challenge in the HiAg and LoAg group. The control group has raised levels of antibodies by 21 dpc as a consequence of the challenge virus.

Antibody levels in the HiAg were >3-fold higher than levels in the LoAg at challenge (Figure 3). Furthermore, antibodies in the HiAg group showed a gradual decline post challenge with the lowest levels being on 21 dpc (Figure 3). Thereafter, antibody levels in the HiAg group were maintained at the same level given that there was no significant difference (*p* > 0.3340) between levels detected at 21 dpc (mean OD_490_ = 4.864 ± SEM = 0.1029, *n* = 12) and 56 dpc (mean OD_490_ = 0.6432 ± SEM = 0.1210, *n* = 12) (Figure 3). In the LoAg group, levels decreased during the incubation period leading to depletion of antibodies by 21 dpc at the onset of mortality. By 56 dpc, antibody levels had not increased above those detected 21 dpc in the LoAg group (Figure 3). In contrast, the control group showed an upward trend of increasing antibody levels between 10 dpc and 56 dpc (Figure 3). High antibody levels maintained during acute infection in the HiAg group (Figure 3) corresponded with delayed onset and low mortalities in this group (Figure 2). On the contrary, the LoAg group which was depleted of antibodies during the acute infection period had an early onset and high mortalities. In summary, these data show that antigen dose corresponded with post immunization antibody levels and PCSP. In addition, high antibody levels (≥ 1.40 OD_490_, Figure 3) prior to challenge could have played an important role in generating the high protection levels obtained in the HiAg group (PCSP ≥ 90%, Figure 2).

### Histopathology and immunohistochemistry (IHC)

**Figure 4:**
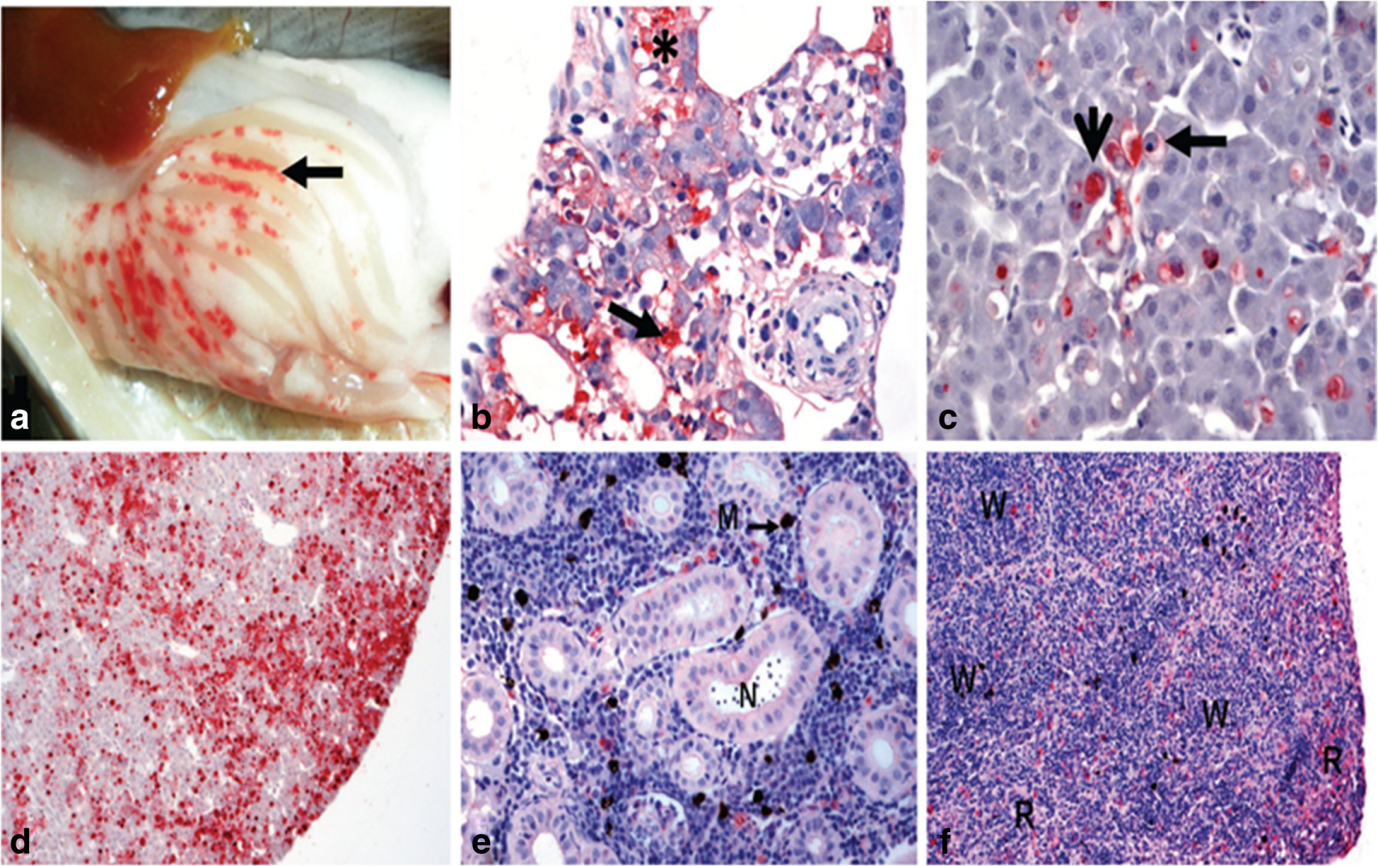
**Pathological changes during the acute stage of infection. a**) Gross picture showing hemorrhages (arrow) in the area of the exocrine pancreas of Atlantic salmon smolt. **b**) Destruction and necrosis of pancreatic acinar cells due to IPNV infection (arrow) with pyknotic nuclei (*). **c**) Different stages of cellular degradation and apoptosis (long horizonal arrow) in the liver (vertical arrow). **d**) Extensive distribution of virus in the liver (red coloration). **e**) Virus in interstitial spaces and leukocytes of the excretory kidney. Melanomacrophages (M) appear as dark spots. **f**) Virus in the white (W) and red (R) pulps of the spleen.

During acute infection, viral antigens were only detected in the pancreas, liver, spleen and headkidney tissues of the LoAg and control groups by IHC (Tables 3 and 4) and not in the HiAg group. In the pancreas, gross pathological changes were characterized macroscopically by hemorrhages (Figure 4a). Single acinar cells whose cytoplasm was filled with virus were observed showing different stages of cellular degradation and later apoptosis (Figure 4b). As the infection progressed, there was total destruction of the pancreatic acinar cells. Similarly, infected hepatocyte cytoplasm was repleted with IPNV (Figure 4c and d), and virus infected cells and neighboring cells formed apoptotic bodies. In kidney tissues, virus infected cells were confined to interstitial tissues and intravascular leukocytes (Figure 4e). In the spleen, detection of viral antigens was evenly distributed in the white and red pulp (Figure 4f). There was no virus detected in the heart, pyloric ceca and other parts of the intestines (not shown). As shown in Tables 3 and 4, viral antigens were only detected by IHC in tissues that had copy numbers ≥ 10^6.5^ and as such, no viral antigens were detected by IHC in tissues of the HiAg group that had viral loads with a mean of viral copy numbers ≤ 10^2.6^ during the acute stage of infection.

**Table 3:** Number of fish detected with virus by IHC and qPCR at 21 days post challenge

**Vaccine group**	**Tissue**	** IHC**		** qPCR**		Log of viral copy numbers in tissues detected by IHC ( Pathology* )
		**No. of infected fish**	**%**	**No. of infected fish**	**%**	
Control	Headkidney	3/6	50.0%	6/10	60.0%	7.51, 7.66, 7.22
	Pancreas	3/6	50.0%	10/10	100.0%	***8.11,8.52, 7.71***
	Liver	3/6	50.0%	10/10	100.0%	***7.35, 8.72, 7.29***
	Spleen	3/6	50.0%	5/10	50.0%	6.54
	Gills	2/6	33.3%	ND	-	-
	Muscle	3/6	50.0%	ND	-	-
	Heart	0/6	00.0%	ND	-	-
	Pyloric ceca	0/6	00.0%	ND	-	-
LoAg	Headkidney	3/6	50.0%	10/10	100.0%	7.64, 7.88, 7.52
	Pancreas	3/6	50.0%	10/10	100.0%	***8.99, 9.07, 8.99***
	Liver	3/6	50.0%	10/10	100.0%	***8.35, 9.02, 9.03***
	Spleen	3/6	50.0%	10/10	100.0%	8.21, 8.05, 8.65
	Gills	0/6	00.0%	ND	-	-
	Muscle	2/6	33.3%	ND	-	-
	Heart	0/6	00.0%	ND	-	-
	Pyloric ceca	0/6	00.0%	ND	-	-
HiAg	Headkidney	0/6	00.0%	10/10	100.0%	-
	Pancreas	0/6	00.0%	10/10	100.0%	-
	Liver	0/6	00.0%	4/10	40.0%	-
	Spleen	0/6	00.0%	10/10	100.0%	-
	Gills	0/6	00.0%	ND	-	-
	Muscle	0/6	00.0%	ND	-	-
	Heart	0/6	00.0%	ND	-	-
	Pyloric ceca	0/6	00.0%	ND	-	-

ND = not done *Tissue detected with pathological changes are shown in *italics.*

**Table 4:** Number of fish detected with virus by IHC and qPCR at 56 days post challenge

**Vaccine group**	**Tissue**	** IHC**		** qPCR**		Log of viral copy numbers in tissues detected by IHC ( Pathology *)
		**No. of infected fish**	**%**	**No. of infected fish**	**%**	
Control	Headkidney	3/6	50.0%	10/10	100.0%	6.78, 6.52, 6.55
	Pancreas	3/6	50.0%	10/10	100.0%	6.78, 6.54, ***7.12***
	Liver	3/6	50.0%	10/10	100.0%	6.57, 6.52, 6.72
	Spleen	3/6	50.0%	10/10	100.0%	6.93, 6.85, 6.51
	Gills	2/6	33.3%	ND	-	-
	Muscle	2/6	33.3%	ND	-	-
	Heart	0/6	00.0%	ND	-	-
	Pyloric ceca	0/6	00.0%	ND	-	-
LoAg	Headkidney	3/6	50.0%	10/10	100.0%	6.78, 6.62, 6.55
	Pancreas	3/6	50.0%	10/10	100.0%	***7.18***, 6.54, ***7.12†***
	Liver	3/6	50.0%	8/10	80.0%	6.57, ***7.22†,*** 6.72
	Spleen	3/6	50.0%	10/10	100.0%	6.93, 6.85, 6.51
	Gills	0/6	00.0%	ND	-	-
	Muscle	2/6	33.3%	ND	-	-
	Heart	0/6	00.0%	ND	-	-
	Pyloric ceca	0/6	00.0%	ND	-	-
HiAg	Headkidney	0/6	00.0%	10/10	100.0%	-
	Pancreas	0/6	00.0%	9/10	90.0%	-
	Liver	0/6	00.0%	6/10	60.0%	-
	Spleen	0/6	00.0%	10/10	100.0%	-
	Gills	0/6	00.0%	ND	-	-
	Muscle	0/6	00.0%	ND	-	-
	Heart	0/6	00.0%	ND	-	-
	Pyloric ceca	0/6	00.0%	ND	-	-

ND = not done *Tissue detected with pathological changes are shown in *italics*.

*†*Lowest log of viral copy numbers at which pathology was detected in the infected organs.

### Infection rates as determined by qPCR

**Figure 5:**
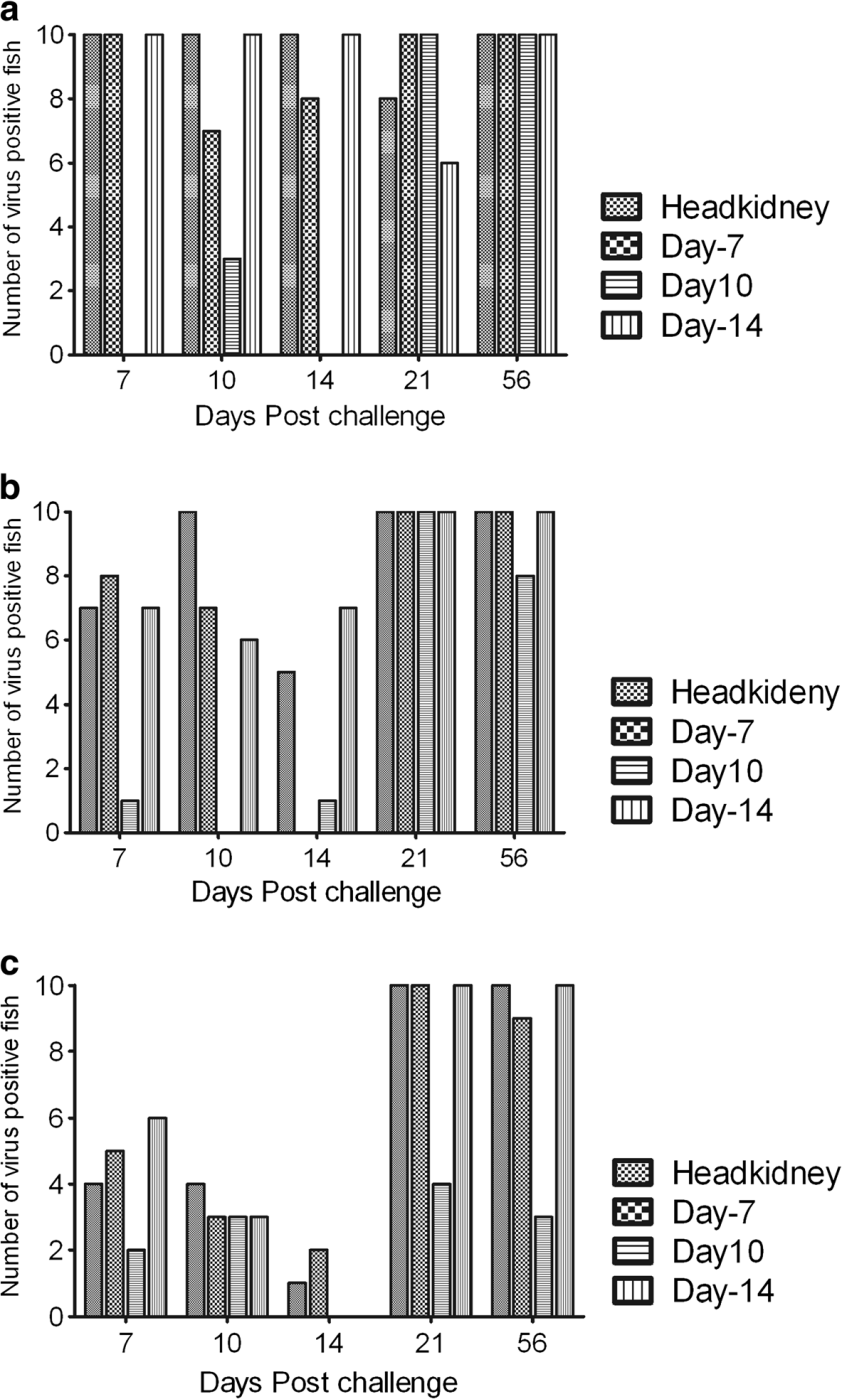
**Number of fish infected with IPNV in different organs detected by qPCR.** Number of infected fish in controls (**a**), LoAg (**b**) and HiAg (**c**) groups at different days post challenge (dpc) up to 56 dpc. *N* = 10 for all groups at all time-points.

The four most infected organs as assessed by IHC (Tables 3 and 4) were used to determine infection rates (morbidity) and to compare differences in tissue susceptibility by qPCR. During the incubation period (7 dpc), the overall infection rate in the control group showed high infection rates reaching up to 100% (Figure 5a). The LoAg group (Figure 5b) had higher infection rates than the HiAg group (Figure 5c). Generally, there was a gradient of infection rates with the highest being in the control group, followed by the LoAg group while fish in the HiAg were less infected during the incubation period (Figure 5a-c). Comparison of tissue susceptibility showed that the pancreas, spleen and headkidney had higher infection rates than the liver in all groups with the highest infections found in the controls (30%, 7 dpc) (Figure 5a-c), followed by the LoAg (10%, 7 and 14 dpc).

During the acute stage (21 dpc), the overall infection rates increased in all groups reaching 100% (*n* = 10) (Figure 5a-c). Comparison of tissue susceptibility showed that infection rates in the liver of the control fish had reached 100% by 21 dpc which corresponded with early onset of mortality in this group (19 dpc, Figure 2). Although infection rates increased to 100% in the spleen, pancreas, and headkidney in the HiAg group, the liver was less infected (≤ 40%, Figure 5c). Hence, the liver was the organ with the lowest infection rates in the HiAg group during acute infection.

In summary, these data show that IPNV has a high tropism for the headkidney, spleen and pancreas during the incubation period. Infection rates increased in these organs during the acute stage. Liver infection and pathology during acute infection in the less protected groups coincided with the onset of mortality. The HiAg group did not show any liver infection in 60% of the fish examined during the acute stage (21 dpc; Figure 5c).

### Quantification of virus by qPCR and correlate of pathology

**Figure 6:**
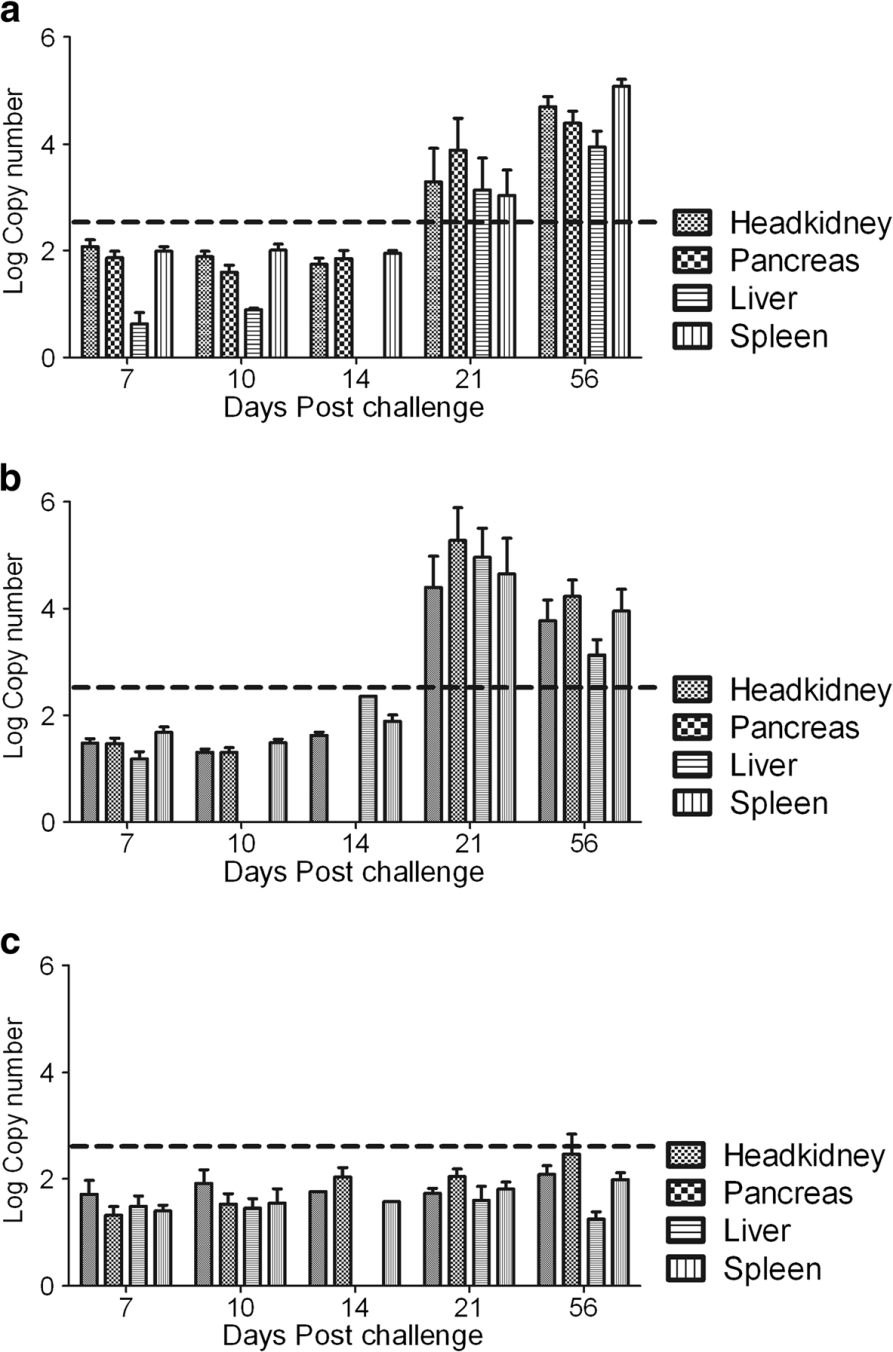
**Relative quantification of virus using qPCR in the infected organs.** Bars show the mean of the log of viral copy numbers in headkidney, pancreas, liver and spleen of the controls (**a**), in LoAg (**b**) and HiAg (**c**) groups at different times post challenge. The control group shows a high level of virus already by 7 dpc increasing up to 56 dpc while the LoAg group shows a marked increase by 21 dpc. The HiAg fish have low viral load throughout the observation period (*n* = 12 ± SEM). Labeling is the same in all figures.

Viral copy numbers detected by qPCR for the four most susceptible tissues detected by IHC (Tables 3 and 4) are shown in Figure 6a-c. During the incubation period (7 dpc), there was no significant difference in viral copy numbers between the vaccinated and control fish (Figure 6a-c)**.** The upper limit of the mean viral copy numbers for all groups was estimated at 10^2.6^ (Figure 6a-c). The liver had lower viral copy numbers than the other organs during the incubation period. During acute infection (21 dpc), the LoAg and control groups had higher viral copy numbers in all organs than the levels detected during the incubation period (Figure 6a-c). In contrast, the mean viral copy numbers in infected organs of the HiAg group were low and did not increase above levels detected during the incubation period (Figure 6c). For example, there was no significant difference between the mean viral copy numbers detected in the headkidney (*p* = 0.9398) at 7 dpc (mean OD_490_ 1.710 ± SEM 0.2624) compared to 21 dpc (mean OD_490_ 1.727 ± SEM 0.09661) and for the liver (*p* = 0.3362) at 7 dpc (mean OD_490_ 1.485 ± SEM 0.1950) compared to 21 dpc (mean OD_490_ 1.245 ± SEM 0.1374) suggesting that levels of viral copy numbers detected in the HiAg group during the incubation period and acute stage of infection were the same. Similarly, there was no significant difference between the mean viral copy numbers detected in the pancreas (*p* = 0.1024) at 10 dpc (mean OD_490_ 1.523 ± SEM 0.1980) compared to 21 dpc (mean OD_490_ 2.040 ± SEM 0.1453) and for the spleen (*p* = 0.3794) at 10 dpc (mean OD_490_ 1.523 ± SEM 0.1980) compared to 21 dpc (mean OD_490_ 1.805 ± SEM 0.1354), further confirming our observation that viral copy numbers in the HiAg group did not increase during the acute stage of infection but remained at the same levels as those detected during the incubation period. Put together, data in Figures 3 and 6 suggest that high antibody levels maintained after challenge in the HiAg group could have played a role in the maintenance of viral copy numbers at the same low level as detected during the incubation period. On contrast, depletion of antibodies during acute infection in the LoAg group corresponded to an increase in viral copy numbers to levels above those detected during the incubation period.

To examine for a possible correlation between virus replication levels and pathology, we determined the quantity of viral copy numbers in organs that had tissue damage as observed by histology and IHC. The lowest viral copy numbers at which tissue damage was observed in the liver and pancreas was equivalent to 10^7.22^ and 10^7.12^, respectively (Tables 3 and 4). All fish with viral copy numbers above these cutoff points had tissue damage. These data suggest that viral copy number ≥ 10^7.0^ correlated with tissue damage in target organs and that this endpoint could serve as a correlate of pathology. It is worth noting that low antibody levels detected in the LoAg and control groups corresponded with viral copy number ≥ 10^7.0^ which in turn correlated with tissue damage in the target organs. In contrast, high antibody levels in the HiAg group corresponded with low viral loads that corresponded with the absence of tissue damage in the target organs. Another important difference between the HiAg group compared to the LoAg and control groups is that liver pathology corresponded with high mortality in the LoAg and control groups. These findings suggest there is a correlation between protective immunity and protection against development of pathology in target organs. Furthermore, 1.4 OD_490_ antibody levels corresponded to the absence of tissue damage in the HiAg group (Figure 3).

### Virus re-isolation

Given that IHC and RT- PCR are indirect methods of measuring virus presence , we included examination of live virus using standard cell culture and show that the control groups had infective virus from headkidney samples during the incubation period (already from 4 dpc, Table 5). The presence of antibodies in the vaccinated groups (HiAg and LoAg) corresponded to the absence of virus from headkidney and blood samples during the incubation period. During the acute stage (21 dpc), infection rates were higher in the LoAg and control groups than in the HiAg group (Table 5).

**Table 5:** Virus re-isolation on RTG-2 cells from headkidney and blood samples

**Sample**	**Vaccine**	**Number of fish detected with virus per timepoint (12 fish were examined at each timepoint)**						
		**0**	**4**	**7**	**10**	**14**	**21**	**56**
Headkidney	HiAg	0	0	0	0	0	1	7
	LoAg	0	0	0	0	0	9	11
	Control	0	12	5	9	5	10	11

### Expression of IFNα and Mx

**Figure 7:**
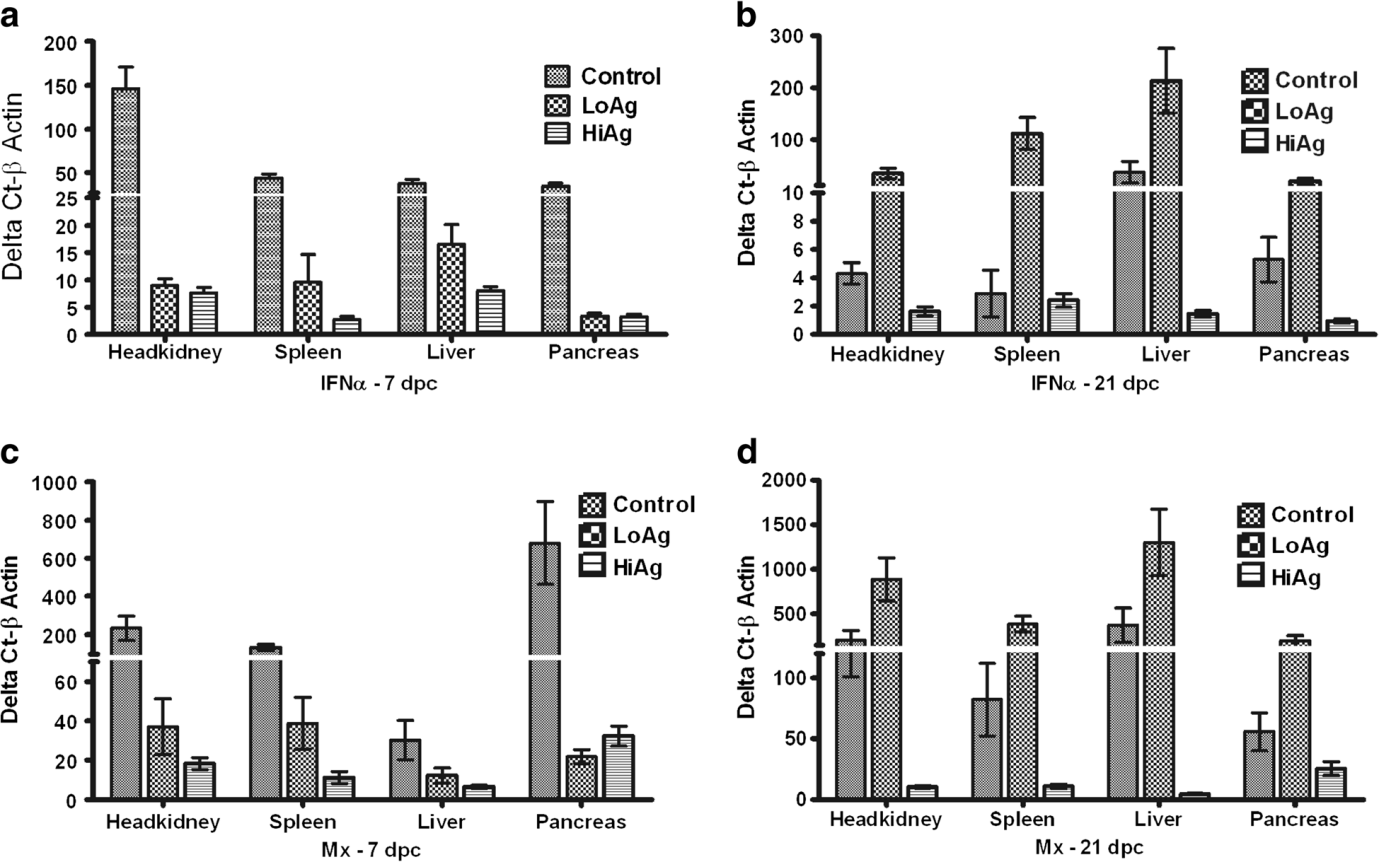
**Expression of IFNα and Mx detected by real time PCR from different organs.** (**a**) IFNα 7 dpc (**b**) FNα 21 dpc (**c**) Mx 7 dpc and (**d**) Mx 21 dpc in the headkidney, spleen, liver and pancreas of controls, LoAg and HiAg groups. IFNα and Mx expression correlates with viral infection and is the highest in the controls at 7 dpc. By 21 dpc, the LoAg group shows the highest expression of both genes in all organs. *N* = 10–12, ± SEM.

Expression levels of IFNα and Mx during the incubation and acute stages of infection are shown in Figure 7a-d. During the incubation period, the control group showed expression of IFNα (Figure 7a) and Mx (Figure 7c) in all virus infected tissues while expression of these genes was low in both vaccinated groups. The highest levels of IFNα were in the headkidney (Figure 7a) while Mx was the highest in the pancreas (Figure 7c). The liver with lower infection rate had poor correlates of viral infection with IFNα and Mx during the incubation period, r^2^ = 0.27 and r^2^ = −0.18, respectively (Table 6).

**Table 6:** Correlates of viral infection in the target organs during the incubation and acute stages of infection

**Time Point**	**Target gene**	**Correlation of gene expression with viral loads in different genes**							
		**Headkidney**		**Spleen**		**Liver**		**Pancreas**	
		**R**^**2**^	***P*-value**	**R**^**2**^	***P*-value**	**R**^**2**^	***P*-value**	**R**^**2**^	***P*-value**
7 dpc	IFNα	0.89	*P* < 0.0000	0.52	*P* < 0.0006	0.27	*P* < 0.2670	0.54	*P* < 0.0733
	Mx	0.72	*P* < 0.0000	0.62	*P* < 0.0001	−0.18	*P* < 0.3059	0.59	*P* < 0.0002
21 dpc	IFNα	0.66	*P* < 0.0000	0.42	*P* < 0.0132	0.75	*P* < 0.0002	0.43	*P* < 0.0081
	Mx	0.69	*P* < 0.0000	0.41	*P* < 0.0160	0.78	*P* < 0.0000	0.40	*P* < 0.0163

During the acute stage (21 dpc) there was a systematic trend that showed that viral loads correlated with IFNα (r^2^ ranging from 0.42 to 0.75) and Mx (r^2^ ranging from 0.40 to 0.78) expression (Table 6). In detail, the LoAg with the highest viral copy numbers in the liver at 21 dpc had the highest levels of IFNα (Figure 7b) and Mx (Figure 7d) and the control group, which had moderately high viral loads, had lower levels of IFNα and Mx. The HiAg group, which had low viral copy numbers in the liver by 21 dpc (Figure 6c), had insignificant expression levels of both genes (Figure 7b and d). Put together, the data in Figures 6 and 7 show that expression of IFNα and Mx correlated with increase in viral loads (Table 6).

## Discussion

One of the greatest challenges in vaccine development is to optimize antigen dose in order to generate a threshold of antibody titer that can be used as a measure of protective immunity. Unlike studies done in humans where challenge models are often not applicable for ethical reasons [33], in this study we show that the antigen dose used in vaccine formulation corresponded with PCSP in vaccinated fish after challenge with IPNV. This demonstrates that it is possible to establish a threshold of antigen dose required to protect fish from mortality that would serve as a measure of vaccine efficacy. In addition, antibody levels obtained after vaccination but prior to challenge corresponded with PCSP, further augmenting the possibility of determining a threshold end point of antibody titer that can serve as a signature of protective immunity. This was in line with assertions made by Pulendran et al. [34] that when a protective threshold of antibody titer is achieved or exceeded, vaccination is assumed to have reached a signature of protective immunity. In higher vertebrates, these tests have been standardized so that signatures of protective immunity based on thresholds of neutralizing antibodies have been determined for different pathogens [35]. However, no similar threshold units of antibody titers have been established in teleosts. In the present study, an antigen dose of 2 × 10^10^ TCID_50_/mL yielding a mean antibody titer ≥ 1.4 OD_490_ at challenge attained a PCSP ≥ 90% after challenge with the highly virulent Norwegian Sp strain NVI-015 in the HiAg group. Therefore these findings demonstrate that at these threshold units of antigen dose (2 × 10^10^ TCID_50_/mL) and antibody titer (≥ 1.4 OD_490_) vaccination against IPNV infection attained high protection (PCSP > 90%) in vaccinated fish. However, it is interesting to note that reducing the threshold unit of antigen dose by one log (10^1^), lowered the antibody titer by threefold and decreased protection to less than half (PCSP ≤ 42%) as shown in the LoAg group that was vaccinated with an antigen dose one log lower than the HiAg group. Overall, these findings demonstrate that in vaccinology, a suboptimal antigen dose one log (10^1^) lower than the protective threshold unit can lead to significant differences in vaccine protection.

Progression of antibody responses for fish used in this study can be divided into two stages namely the immune induction and post challenge periods. Immune induction covered the period between vaccination up to the time of challenge at 8 wpv in which fish developed antibodies in response to vaccination. At challenge (8 wpv), antibody levels in the HiAg were threefold higher than levels in the LoAg suggesting that post vaccination antibody responses were antigen dose dependent. The post challenge period covered the incubation and acute stages of infections. During the incubation period, antibody levels in both the HiAg and LoAg groups declined, which could be attributed to their utilization in virus neutralization after challenge. Neutralization of virus by antibodies could have played a major role in delaying the onset of post challenge mortality in vaccinated fish unlike the control group in which no antibodies were present to neutralize replicating virus; fish therefore started dying by 19 dpc. It is likely that mortality in the LoAg group was delayed until after depletion of antibodies which gave way to an increase in virus replication by 21 dpc. High antibody levels in the HiAg group could have played a major role in suppressing the increase in virus replication during the acute stage of infection leading to delayed onset and reduction of mortality in this group. While antibodies in the vaccinated groups declined due to their usage in virus neutralization, control fish that had no antibodies prior to challenge developed antibody responses that increased to higher levels during the acute stage of infection as an indication of a primary response of exposure to the challenge virus. This could explain reasons why the LoAg group had lower antibody levels than the control group during acute infection (21–56 dpc). The major difference between the vaccinated and control fish is that antibodies from vaccinated fish were generated in response to vaccination from inactivated vaccines prior to challenge while antibodies from control fish were generated in response to the challenge virus. Hence, antibodies from control fish could not have played a major role in post challenge protection because they were generated after exposure to the challenge virus. On the contrary, antibodies from vaccinated fish could have played a major role in post challenge protection because they were generated prior to challenge from inactivated vaccines for protection against the challenge virus.

One of the prime objectives in vaccination is to prevent establishment of infection soon after exposure to the virus. Overall infection rates of the current study were higher in control fish during the incubation period, followed by the LoAg group that had low antibody levels with the lowest infection rates being in the HiAg that had the highest antibody levels. This was consistent with our earlier observations that fish vaccinated with less potent vaccines get infected much earlier with higher infection rates than fish vaccinated with more potent vaccines [11]. Given that increase in antibody levels corresponded with decrease in overall infection rates, it is likely that circulating antibody levels played an important role in preventing dissemination and seeding of virus in the predilection sites in the HiAg group. This was supported by virus re-isolation data in which the number of fish having viraemia was the lowest in the HiAg group and was consistent with our previous study in which we showed that high antibody levels were correlated with the absence of viraemia, unlike the less protective vaccines that had early onset of viraemia and linked to high infection rates [11]. Another important observation made from this study was that although the pancreas and liver are target organs prone to tissue damage, the seeding of virus in the liver was delayed until the acute stage of infection for most fish while infections in the pancreas occurred early during the incubation period. Furthermore, these data show that for fish that were only infected in the pancreas without involvement of the liver, infection did not proceed to cause pathological disease. These findings point to the importance of involvement of the liver during acute infection. This was clearly demonstrated in the HiAg group that had low infection rates in the liver and yet the pancreas had 100% infection rates, but because the liver was not infected in most fish, no pathological disease developed. Consequently, the seeding of virus coupled with tissue damage in the liver corresponded with an increase in post challenge mortality in the LoAg and control groups during acute infection. Conversely, the presence of high antibody levels in the HiAg group corresponded with low infection rates in the liver. Although we did not determine the role of the cellular mediated immune response in eliminating virus infected cells, these observations suggest that high antibody levels could play an important role in preventing the seeding of virus in the liver as a protective mechanism against tissue damage. This was in line with observations made elsewhere that preventing deposition of virus in target organs prevents the establishment of clinical disease [36,37]. However, it is important to point out that it is possible that other arms of the immune system not evaluated in the present study could have contributed to reducing post challenge mortality. This was demonstrated in the LoAg group that had lower antibody levels and higher viral copy numbers at 21 dpc than the control group and yet this group had less mortality than the control group.

As pointed out by Plotkin [35] the correlate of clinical disease is not necessarily the same correlate that predicts the cut-off against infection. In the present study, infection was detected in tissues with mean viral copy numbers ≤ 10^2.6^ which corresponded with the expression of innate immune genes. However, these viral copy numbers were inadequate to cause tissue damage in target organs. It is only when viral copy numbers exceeded 10^7.0^ that tissue damage was detected, implying that this cutoff could serve as a correlate of pathology in target organs. By determining the quantity of viral copy numbers required to establish pathological disease, immunization can be optimized to obtain antibody levels able to prevent tissue damage. For example, in the case of measles vaccines [38], antibody titers > 200 mIU/mL were protective against infection, whereas titers between 120–200 mIU/mL were protective against clinical disease but not infection. Titers <120 mIU/mL were not protective at all. Although we did not determine the cut-off that prevents establishment of infection, our findings show that at 1.4 OD_490_, antibody levels corresponded with the absence of tissue damage in the HiAg group. In terms of tissue susceptibility, data presented here show that limiting virus replication in the liver and pancreas corresponded with failure to establish pathological disease during acute infection. This was shown in the HiAg group that had low viral copy numbers linked to the absence of tissue damage. Conversely, copy numbers ≥ 10^7.0^ in the liver and pancreas of the LoAg and control groups were linked to tissue damage and high mortality. Although we did not rule out the possible involvement of the cellular mediated immune response in eliminating virus infected cells, these data suggest that antibodies could play a pivotal role in suppressing virus replication to levels below the correlate of pathology as a mode of protection against tissue damage and mortality.

Innate immune genes like IFN and IFN-inducible genes (IGS) are known to be the first line of defense against viral infections [39-41]. Expression of IFNα and Mx correlated with establishment of infection in the control group during the incubation period indicating that upon infection, IPNV induces the expression of early immune genes. Expression of these genes did not only correlate with infection at predilection sites, but was also highly correlated with an increase in viral copy numbers suggesting that these genes could serve as biomarkers of tissue tropism as well as indicators of increase in virus replication. This entails that, instead of tracking virus distribution and monitoring increase in replication capacity, expression of these genes could serve as alternative indicators of tissue tropism and an increase in viral copy numbers. For example, high viral loads in the liver during acute infection correlated with high expression levels of both genes while low infection rates during the incubation period were characterized by low expression levels. In vaccinated fish, up-regulation of these genes was indicative of low efficacy or vaccine failure as shown in the LoAg group in which an increase in viral copy number during acute infection was marked with high levels of IFNα and Mx. Conversely, down regulation of these genes in the HiAg was indicative of high protection linked to low viral copy numbers. Here, we demonstrate for the time, that the kinetics of IFNα and Mx expression follow the distribution and replication of IPNV making these genes potential biomarkers of infection progression as well as indicators of vaccine efficacy.

In summary, this study has shown that the mechanism of vaccine protection against IPNV infection is a stepwise process in which vaccine induced protection reduces post challenge infection rates as a first step. This implies that some fish do not get infected and for fish that get infected, the next mode of protection is to prevent seeding of virus in target organs, particularly in the liver. Finally, for fish that get infected in target organs, protection is by reducing virus replication to levels below the correlate of pathology. Determining the correlate of pathology based on viral copy numbers at which tissue damage is established provides a benchmark against which vaccine protection can be measured. Use of innate immune genes as indicators of tissue tropism and increase in virus replication provide the first methodological evidence that gene signatures can be used as biomarkers of IPNV infection progression in salmonids. We demonstrate that antigen dose in vaccine formulation corresponds with vaccine efficacy and that antibody levels can be used as a signature of protective immunity against pathological disease and mortality. Overall, these findings show that by streamlining testing procedures, immunological thresholds that reliably protect fish from disease can be established in IPNV vaccinology.

## Abbreviations

LoAg: Low antigen; HiAg: High antigen; PCSP: Post challenge survival proportions; dpc: Days post challenge; dpc: Days post vaccination; RT = PCR: Real time polymerase chain reaction; IHC: Immunohistochemistry; IPNV: Infectious pancreatic necrosis virus; qPCR: Quantitative polymerase chain reaction.

## Competing interests

The authors declare that they have no competing interests.

## Authors’ contributions

ØE, HMM, SM, RAD: conceived and designed the study. HMM, BNF, SM: carried out efficacy trials and generated laboratory data. HMM, ØE, SM: data analysis and preparation of the manuscript. ØE, RAD: acquisition of resources. All authors read and approved the final manuscript.
